# The Influence of Local Constraints on Solvent Motion in Polymer Materials

**DOI:** 10.3390/ma17194711

**Published:** 2024-09-25

**Authors:** Krzysztof Hałagan, Przemysław Duniec, Marcin Kozanecki, Andrzej Sikorski

**Affiliations:** 1 Department of Molecular Physics, Faculty of Chemistry, Lodz University of Technology, Zeromskiego 116, 90-543 Lodz, Poland; krzysztof.halagan@p.lodz.pl (K.H.);; 2 Institute of Physics, Lodz University of Technology, Wolczanska 217/221, 93-005 Lodz, Poland; 3 Faculty of Chemistry, University of Warsaw, Pasteura 1, 02-093 Warsaw, Poland

**Keywords:** copolymerization, dynamic lattice liquid, Monte Carlo method, polymer solutions, solvent dynamics

## Abstract

The influence of obstacles in the form of polymer chains on the diffusion of a low-molecular-weight solvent was the subject of this research. Studies were performed by computer simulations. A Monte Carlo model—the Dynamic Lattice Liquid algorithm—based on the idea of cooperative movements was used. The tested materials were polymer networks with an ideal structure (with a uniform mesh size) and real, irregular networks (with a non-uniform mesh size) obtained numerically by copolymerization. The diffusion of the solvent was analyzed in systems with a polymer concentration that did not exceed 16%. The influence of the polymer concentration and macromolecular architecture structure on the mobility and character of the motion of the solvent was discussed. The influence of irregular network morphology on solvent dynamics appeared to be significantly stronger than that of regular networks and star-like polymers.

## 1. Introduction

The motion of small particles including solvent molecules in polymer solutions, melts, and networks has been extensively studied by means of gravimetry, membrane permeation, fluorescence, dynamic light scattering, inverse gas chromatography, nuclear magnetic resonance spectroscopy, fluctuation correlation spectroscopy, and depolarized dynamic light scattering showing the influence of polymer concentration and chain length on the mobility of moving objects [1,2,3,4,5,6,7,8,9,10,11,12,13,14]. Experiments have shown a plethora of dynamic behaviors in complex macromolecular systems [15,16,17,18,19,20,21]. In many cases, subdiffusive behavior has been found, which means that the Einstein–Smoluchowski equation is not fulfilled and that the mean square displacement of moving objects scaled with time as *t^α^* with *α* varies between 0.2 and 0.9 [16,22]. The non-Fickian diffusive behavior of diffusants in macromolecular systems has also been predicted by theories [23,24,25]. These theories are based on obstruction effects, where the positions of polymer chains are fixed. This approach gives correct results for small diffusants in dilute and semi-dilute solutions [26,27,28,29,30], for considering hydrodynamic interactions [31,32,33,34], or for an assumption that free volume is a key factor controlling the mobility of diffusants [35,36,37,38,39,40].

Simulations of coarse-grained dynamics models in some dense polymer systems have recently been carried out [41,42,43,44,45,46,47,48]. In this research, the model system was represented by random walks without excluded volume (Gaussian chains), and the conformations of these macromolecules remained unchanged; i.e., the time scales of polymers and diffusants were split. The simulations carried out within the frame of the Dynamic Lattice Liquid (DLL) model revealed a subdiffusive motion. The mobility of non-bounded small diffusants was found to be weakly dependent on chain mass and polymer concentration [42,43]. Simulations of motion in dense macromolecular systems have been carried out using the Molecular Dynamics technique, using coarse-grained and atomistic models, and the macromolecular environment was treated as a cluster of obstacles formed of chains, although most studies have focused on the influence of the diffusant size on its motion [48,49,50,51,52,53,54,55,56,57,58,59,60,61,62]. It was also shown by means of Discontinuous Molecular Dynamics simulations that the structure of the matrix formed by obstacles has an influence on diffusion, although the differences in mobility were found to not be large, and a common scaling behavior was confirmed for systems consisting of small objects and chains [54].

The picture of object mobility in polymer systems is now well established, but the study of highly cross-linked networks is still computationally poorly accessible. Thus, in this paper, a study of the motion of solvent molecules comparable in size to the monomer unit size is presented. This study covered several polymer systems—regular polymer stars (which can be considered a model of a single crosslink point in a network) and regular and irregular networks. The main purpose of this presented study was to show the difference in the dynamic behavior of a solvent for regular polymer networks, as usually presented in polymer textbooks, versus real polymer networks obtained by the copolymerization reaction for polymer materials. There was an important difference between the studied treatment and other lattice models generally based on the ‘ant in the labyrinth’ concept [22]. In the latter kind of model, a single object travels on a lattice, jumping from one vacancy onto another (the vacancies are usually regarded as fluctuating free volume, and their size is comparable with that of the mobile elements). In these kinds of models, neither correlations nor hydrodynamic effects are assumed. In the model presented, correlations in motion between movable elements were taken into consideration, both based on using the cooperative motion of elements and hydrodynamics, which are apparently crucial for diffusion in a macromolecular crowded environment [5,63,64,65]. This model was simulated using the DLL technique, which was previously applied in studies of dynamics in complex dense macromolecular systems [66,67,68].

## 2. Materials and Methods

A coarse-grained model of linear polymers, represented as beads connected by non-breakable bonds, was developed. The macromolecular system under consideration was composed of flexible chains immersed in an explicit solvent. The solvent was assumed to be monomeric, and the solvent molecules were of the size of a single polymer bead (single mer). The objects in the DLL model, i.e., solvent molecules and repeating units of polymer, were embedded in a face-centered cubic lattice structure with all lattice sites in the system occupied by polymer beads or solvent molecules. The system was athermal, that is, the excluded volume was the only potential of interaction used. It has been shown that the properties of the system obtained within the DLL model correctly reflected the dynamic behavior in various polymer systems [43,46,69,70,71]. It was also assumed that the system had some excess volume, and thus each object would only have enough space to vibrate around its position as defined by the lattice site. However, the system is dense, and objects cannot move easily over larger distances to other lattice sites because all sites are occupied. However, in such dense systems, long-range mobility can be realized using the DLL algorithm, in which object translations are performed collectively over distances exceeding the vibration range. Each displacement of an object from its mean position is assumed to be an attempt of movement to a neighboring lattice site. The directions of movement attempts are allowed along vectors connecting neighboring lattice sites and, therefore, are randomly distributed among the *q* directions, where *q* = 12 is the lattice coordination number. Attempts are considered successful if they coincide in such a way that the sum of displacements is close to zero (condition of continuity) along a path including more than two molecules. All of the objects that do not contribute to correlated sequences (circuits) satisfying this continuity condition are non-moveable at a given time step.

The model described above has been implemented as a dynamic Monte Carlo simulation algorithm for macromolecular systems in a solvent [66,67,68]. A field of randomly chosen unit vectors, which are assigned to objects and point in directions along lattice vectors, represents motion attempts. After setting all vectors leading to unsuccessful attempts to zero, only vectors contributing to closed circuits (loops) remain, and they are treated as traces for possible rearrangements. All possible rearrangements are performed by shifting beads along these closed loop traces, while each bead shifts to a neighboring lattice site. In this algorithm, the following steps can be distinguished: (i) generation of the vector field representing attempts of movement, (ii) elimination of non-successful attempts (including motion resulting in breaking a bond), and (iii) replacement of beads within all closed loops. A single Monte Carlo time step consisted of the procedures described above (i–iii).

The motion of solvent in three different polymer systems was studied. The first system (sample code PM) was a regular three-dimensional polymer network consisting of chains fixed in space (immobile), and, therefore, the dispersity was *Ð* = 1 here. The mesh size (number of monomer units between crosslinks) was exactly 3, 7, or 11 (PM-03, PM-07, and PM-11, respectively). In this case, the network was predefined, and the rest of the system was filled with the mobile solvent. The second system was an irregular network (sample code sPM) synthetized by copolymerization of a 2-functional monomer and a 4-functional crosslinker (the reaction scheme was shown previously in [72]), using the same DLL algorithm, with the average mesh size of 3.0297, 7.1551, and 11.3765 (sPM-03, sPM-07, and sPM-11 respectively). The dispersity concerning the mesh size was 1.441, 1.356, and 1.328 (for sPM-03, sPM-07, and sPM-11, respectively). The initial numbers of the initiator, cross-linker (junctions of the network), and monomer were tuned to obtain the largest polymer cluster (just after reaching the gel point) to have the target concentration as close as possible to PM systems. The initial initiator/crosslinker/monomer molar ratios were taken as 10/30/100, 1/3/40, 1/3/60 for sPM-03, sPM-07, and sPM-11, respectively. The probability of the addition reaction was set to 0.0001. When the reaction was stopped, all molecules, except the largest cluster, were replaced by the solvent, and the main simulation run was started. In this case, all of the elements of the system were able to move. The third system consisted of star-shaped macromolecules (sample code T). The star macromolecule was made up of *f* = 3, 6, and 12 arms, and the core of the star consisted of 1 bead (T_1_1, T_1_2 and T_1_3). Higher numbers of arms were achieved using cores consisting of 3 beads (average *f* = 22.8, T_1_4) and 5 beads (*f* = 32.8, T_1_7). The arm was a flexible linear chain consisting of *m* = 50 beads on average, and the number of star macromolecules varied between 47 (T_1_7) and 494 (T_1_1). The dispersity of arm lengths was 1.0223, 1.0255, 1.0327, 1.0535, and 1.0932 (for T_1_1, T_1_2, T_1_3, T_1_4, and T_1_7, respectively). The star cores were randomly placed in the system, and then core-first polymerization was started (cores were reaction initiators). The addition reaction probability was set to 0.005. The reaction was stopped after reaching the target polymer concentration. Then, all unreacted monomers were replaced by solvent molecules. Additional 10^7^ simulation steps were performed for equilibration of the system prior to the main simulation run. Again, all elements of the system were able to move. No crosslinking was present between stars, so, in this case, the system can be considered an assembly of not-connected network nodes with loose mesh chains. The size of the system, i.e., the Monte Carlo box, was *L* × *L* × *L* = 144^3^ lattice constants (equal to mer size) in all the cases presented. Polymer concentration, *Φ_p_*, can be calculated as the ratio of the number of nodes occupied by the polymer beads to the number of all nodes in the system. In PM systems, *Φ_p_* was 0.15625 (PM-03), 0.04297 (PM-07), and 0.01967 (PM-11). The polymer concentration in the sPM systems was 0.15467 (sPM-03), 0.0411 (aPM-07), and 0.01929 (sPM-11). The polymer concentration in T systems was 0.049 (T_1_1), 0.052 (T_1_2, 0.049 (T_1_3), 0.050 (T_1_4), and 0.051 (T_1_7). These values implied that the polymer concentration was well below the percolation threshold, and therefore the movement of small solvent molecules was not limited, although subdiffusive behavior could be expected [44].

## 3. Results and Discussion

**Figure 1:**
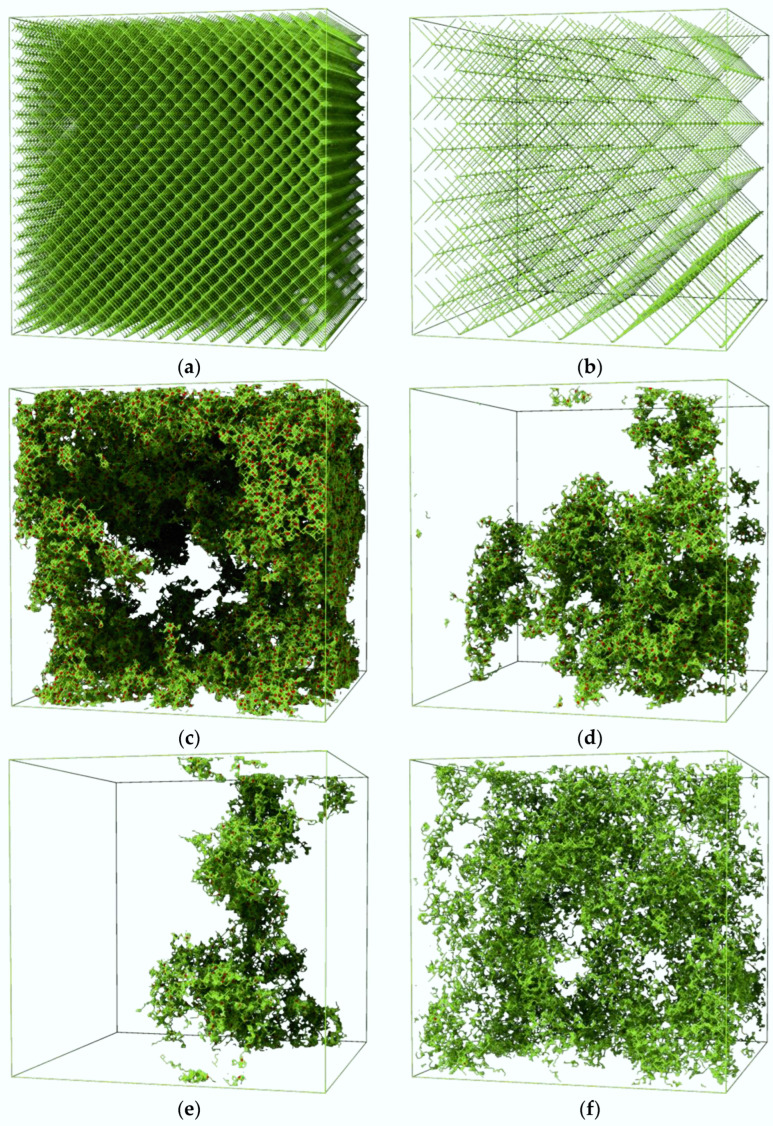
System morphology (only the polymer phase is visible for clarity) in the case of (**a**) PM-03, (**b**) PM-11, (**c**) sPM-03, (**d**) sPM-07, (**e**) sPM-11, and (**f**) T_1_3.

Before presenting how solvent dynamics depend on the structure of macromolecular systems, examples of the polymer systems studied are shown. Figure 1a–f present a visualization of the studied macromolecular systems. While the structure of a regular PM is obvious, in the case of irregular sPM networks, one notices large randomness in the shape of the network and large differences in the local polymer density in the systems, regardless of the average mesh size.

**Figure 2:**
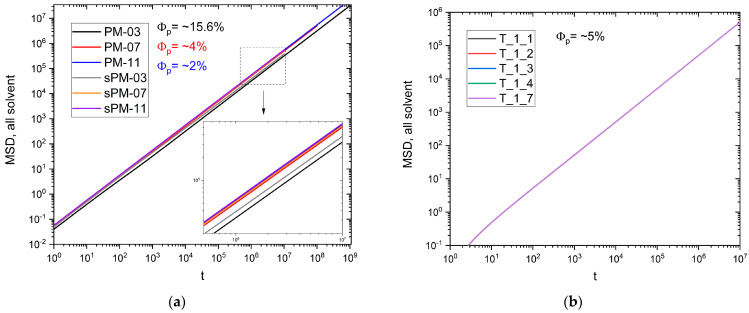
MSD versus time for (**a**) polymer networks and (**b**) star systems. The inset shows the zoom in the dashed region. The polymer concentration values are also given (PM and sPM are similar). The time axis is expressed in Monte Carlo steps.

The first parameter studied is the mean square displacement Δ*r*^2^ (MSD). Figure 2a,b show the dependence of MSD on time, *t*, in a double logarithmic scale for PM, sPM and T systems. The MSD values here were averaged over all solvent molecules in the system. It can be seen that, except for short times, the dependence of MSD values on time is linear. As one might expect, the smaller the mesh size, the smaller the MSD value. The MSD for irregular networks (sPM) is, in all cases, larger than for regular networks (PM), which is visible in Figure 2a. The reason is the existence of large regions of pure solvent where the solvent moves in bulk; this can be clearly seen in Figure 1. For PM samples, solvent movement is more or less restricted in the entire volume. It can also be seen that systems containing star-branched polymers (Figure 2b) show no significant differences in MSD values. Thus, the influence of the local polymer concentration is not found, which, after all, depends significantly on the number of arms in the stars [73] and is determined by the total polymer concentration, which was similar in all systems, T. Furthermore, the MSD for star polymers is almost an order of magnitude higher than for both networks when we compare systems with similar concentrations, namely PM-11 and sPM-11 and T_1_1.

**Figure 3:**
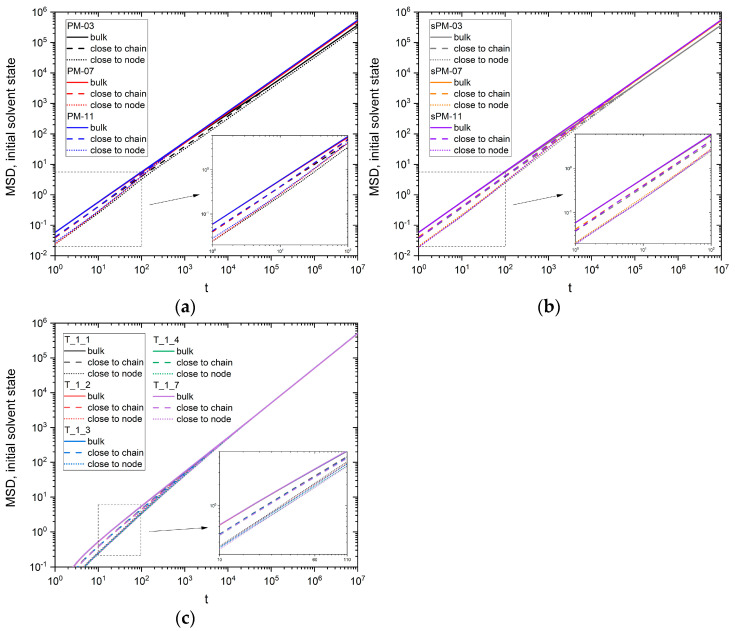
MSD versus time for different fractions of solvent in the cases of (**a**) PM, (**b**) sPM, and (**c**) T systems. The insets show the zoom in the dashed region.

Since most of the solvent at the polymer concentrations studied (between 2% and 15%) does not ‘feel’ the influence of macromolecules, it appears that more information can be obtained from analyzing the movement of the solvent near the polymer elements (beads). Figure 3a–c shows the MSD as a function of time, *t*, for solvent molecules that had one or two contacts (nearest neighbor as polymer unit, called the ‘close to chain’ configuration) and more than two contacts (close to chain or node, called the ‘close to node’ configuration) with the polymer at the initial time (*t* = 0) for all PM, sPM, and T systems studied. The MSD for the remaining solvent in the system (‘bulk’, with no polymer contacts) is given as a reference value in these figures. It can be seen that the dependence of the MSD on time is a power function for all the systems studied. It should be noted that for short times, differences in solvent mobility depending on the mesh size or number of arms are visible. For long time scales in PM and sPM systems, the differences, although smaller, still exist over the entire range studied. In the case of stars, the differences disappear for longer time scales.

**Figure 4:**
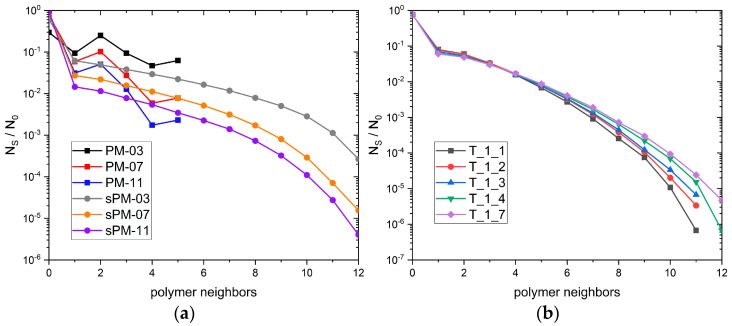
Number of solvent–polymer contacts normalized to the total system size for (**a**) polymer networks and (**b**) star systems.

Next, what the number of polymer–solvent contacts look like if one considers a single solvent molecule was checked. Figure 4a,b show which fraction of solvent molecules *N_s_*/*N*_0_ have on average in a given number of contacts with the polymer for the PM, sPM, and T systems studied. As mentioned above, most of the solvent molecules in the system are not in contact with the polymer at any given time. Of course, the smaller the mesh size, the more solvent molecules interact with polymer chains, and the differences between the PM and sPM systems are more significant. The fraction of solvent molecules that have more contacts with the polymer decreases very rapidly. The local increase in the values of this parameter for two and five are only due to the lattice model used. The dependence of the fraction *N_s_*/*N*_0_ for star-like polymers (Figure 4b) in general looks similar and is caused by statistical errors. In the range of values where the data are reliable, there is no dependence on the number of arms.

The self-diffusion coefficient *D* of a given object in a three-dimensional space can be determined based on the Einstein–Smoluchowski equation as follows:

(1) $$<∆r^{2}>=6Dt$$

**Figure 5:**
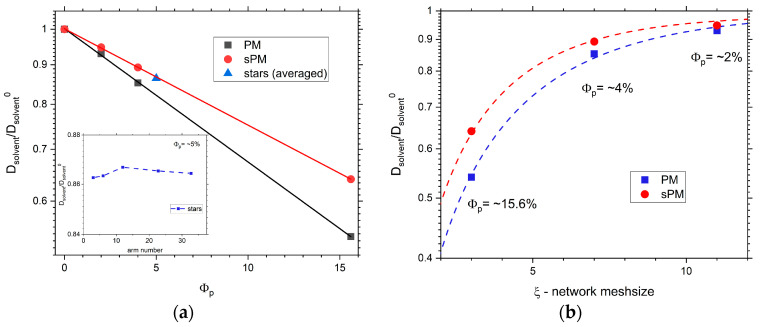
Normalized self-diffusion coefficients for (**a**) PM, sPM, and T systems versus polymer concentration and (**b**) PM and sPM versus network mesh size. The inset on (**a**) shows the dependence of the self-diffusion coefficient on the arm number in the case for T systems. The error bars in all cases are comparable to symbol size. Dashed lines are used only as ‘guide for eyes’.

The determination of the diffusion coefficient for solvent *D* is therefore possible if MSD depends linearly on time. Figure 5a shows the values of the reduced solvent self-diffusion coefficient for all tested systems as a function of polymer concentration. The reduced self-diffusion coefficient of the solvent was calculated as *D_solvent_*/*D_solvent_*^0^, where the subscript 0 indicates a system composed of only the solvent. The values for star-shaped polymers were averaged over all systems tested, as it turned out that the value of the diffusion coefficient hardly depended on the number of arms (see the inset of Figure 5a). The *D_solvent_*/*D_solvent_*^0^ (*ϕ_p_*) relationship is a power function for both regular and irregular networks, and the rate of decreasing the self-diffusion coefficient with polymer concentration is higher for the regular network. The value of the *D_solvent_*/*D_solvent_*^0^ ratio for stars lies on the curve for the sPM system. The inset of Figure 5a shows the dependence of *D_solvent_*/*D_solvent_*^0^ on the number of arms of stars (T system). It can be seen that the changes in the reduced self-diffusion coefficient are not large, although one can point to a small maximum in the value of *D_solvent_*/*D_solvent_*^0^ for the number of arms, *f* = 12. However, it should be noted that, here, one may be dealing with the influence of the structure near the branching point (*f* = 3, 6, and 12 stars have a single-bead core while those with more arms have a core composed of some beads). Figure 5b shows how the value of the reduced self-diffusion coefficient changes as a function of the mesh size for the PM and sPM systems. For dense networks—that is, for a small mesh size—the differences in diffusion coefficient between PM and sPM systems are greater, but they disappear as the mesh size increases. This is because the looser the network, the more similar the solvent dynamics will become to that corresponding to the bulk state.

**Figure 6:**
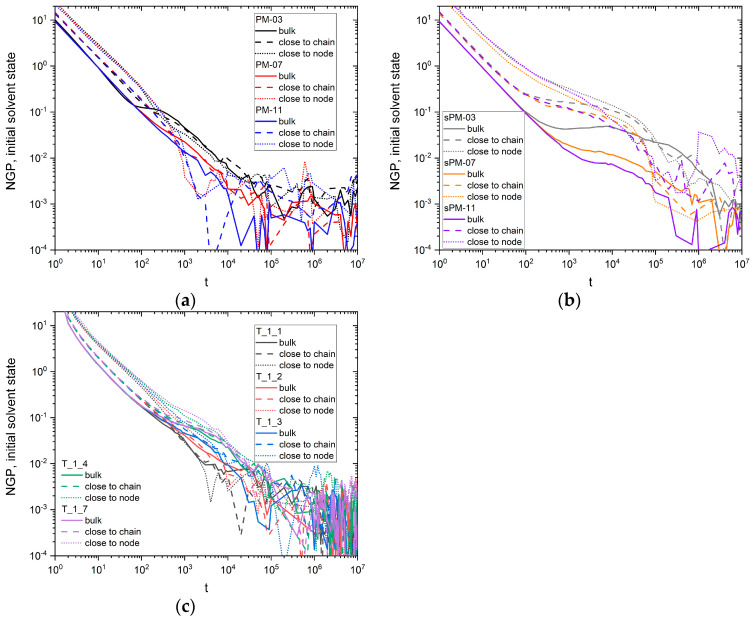
Non-Gaussian parameter (NGP) *α*^2^(*t*) for (**a**) PM, (**b**) sPM, and (**c**) T systems, presented for different fractions of solvent.

**Figure 7:**
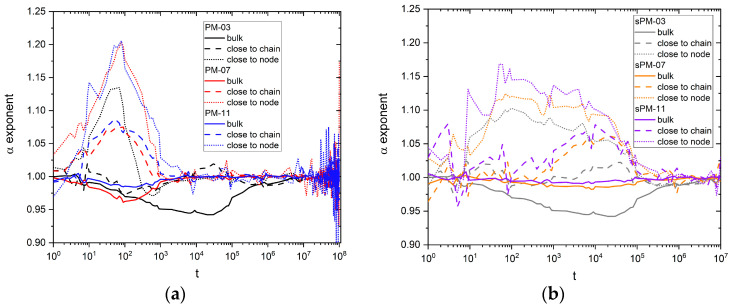
Exponent *α* versus time for various solvent fractions for (**a**) PM and (**b**) sPM systems.

Since it is difficult to determine, based on the MSD analysis of the solvent molecules, if and under what conditions deviations from the Einstein–Smoluchowski equation occur, a non-Gaussian parameter (NGP) *α*^2^(*t*) was examined. It is defined as follows [24]:

(2) $$\alpha^{2}\left(t\right)=\frac{3}{5}\frac{<∆r^{4}>}{{<∆r^{2}>}^{2}}-1$$

where symbol < > represents the ensemble averaging. Figure 6a–c shows the dependence of this parameter on time in all PM, sPM, and T systems studied. The presented data are not perfect from a statistical point of view, so conclusions about subtle changes among systems are not definitive. It can be seen that in all plots the value of this parameter decreases, and there are no significant jumps in the value of *α*^2^(*t*) (those for longer times relate to weak statistics); in a long time limit, the value of *α*^2^(*t*) in sPM systems is clearly higher, and the decrease in value is smoother. From Figure 6a, it can be seen that in the regular PM network, a weaker decrease in *α*^2^(*t*) is observed for low mesh size values, and a faster decrease is observed for high values. Figure 6b shows that, in the irregular sPM network, the trends are clearly stronger. Furthermore, the inflection point can be observed for all samples in this case near 10^4^ time steps. This corresponds to the maximal change in the *α* exponent, as shown in Figure 7b. The decreasing value of *α*^2^ with time is also visible in Figure 6c for T systems, and no correlation with the behavior of this parameter and the number of star arms can be seen there.

The confirmation of the occurrence of subdiffusion, that is, the relationship Δ*r*^2^ ~ *t^α^* with an exponent *α* < 1, can be obtained directly by analyzing the changes in the exponent α of this equation. The best way to determine it is to calculate the logarithmic derivative of the MSD as follows:

(3) $$\alpha =\frac{dlog<∆r^{2}>}{dlog\;t}$$

Figure 7a,b shows the time dependence of the exponent *α* for solvent motion in the PM and sPM systems studied. If one considers only the bulk solvent, the strongest subdiffusion occurs for the lowest values of the mesh size, both for PM and sPM. The behavior of the exponent *α* in the PM system (Figure 7a) for solvent ‘close to node’ and ‘close to chain’ surprisingly shows the appearance of superdiffusion-like behavior for times between 10^1^ and 10^3^ steps for all mesh sizes (classical superdiffusion is the case when *α* > 1). However, here, this is a purely geometrical effect (not a ballistic diffusion); when the solvent leaves the polymer-rich region, it starts to move faster—so there is a positive change in the slope of the MSD, and, consequently, a value of *α* greater than 1, as a mathematical derivative, is observed. This behavior is weakest in the case of the smallest mesh size. For sPM systems (Figure 7b), this phenomenon for ‘close to node’ and ‘close to chain’ occurs over a wider time window (up to 10^5^). These differences are probably due to the different homogeneity of PM and sPM networks. The differences in the local density and homogeneity of the two types of polymer networks are just visible in the example configurations presented in Figure 1. Regions of low and high local polymer density in sPM networks lead to the timing of anomalous diffusion phenomena over a wider time range. In the sPM case, it is more difficult for the solvent to leave regions of higher polymer concentration, and, consequently, this process takes a longer time.

**Figure 8:**
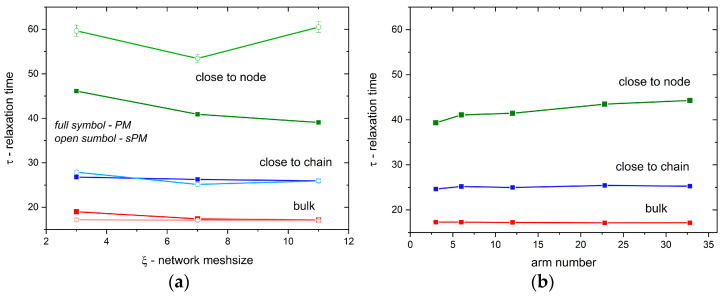
Relaxation times obtained from fitting the autocorrelation functions with the KWW relation for various solvent fractions for (**a**) PM and sPM and (**b**) T systems versus the number of arms in T systems (for the same polymer concentrations).

Another important quantity for analysis is the diffusion relaxation time, which provides the information of the residence time for a given fraction of the solvent. These can be determined from the time-dependent position autocorrelation function *A*(*t*), defined as a change in the solvent position at time *t* relative to its initial position [43]:

(4) $$A(t)=\frac{1}{N_{S}}\sum_{i}\delta_{i}$$

where *N_S_* is the number of solvent molecules in the analyzed solvent fraction, *δ_i_* is equal to 1 if the same solvent molecule occupies the site *i* at time *t* and *t*_0_ = 0, otherwise *δ_i_* = 0. From this function, the relaxation time *τ* can be determined using the stretched exponent dependence formula *A*(*t*) ~ exp((−*t*/*τ*)*^β^*) with *β* as the fitting parameter (in all cases close to 0.8). Figure 8a,b shows the relaxation time for the PM, sPM, and T systems studied as a function of mesh size (networks) or arm number (stars). For both networks, regular and irregular, the relaxation times of solvents ‘in bulk’ and ‘close to chain’ practically do not depend on the mesh size, except that the solvent ‘in bulk’ relaxes faster than the ‘close to chain’ one. The situation changes in the case of the ‘close to node’ solvent; in the regular network, relaxation occurs much faster than in the irregular network (and faster than in the ‘in bulk’ and ‘close to chain’ cases discussed above). Moreover, in the former case, the relaxation time decreases, and the changes are not monotonic (a minimum appears around the mesh size equal to 7 units). For systems containing star-like macromolecules, the relaxation time values are similar, i.e., the ‘close to chain’ solvent relaxes more slowly than the ‘bulk’ one, and they weakly depend on the arm number and are smaller than those corresponding to PM systems. As in the PM and sPM systems, the ‘close to node’ solvent relaxes more slowly, and its relaxation time slowly increases with the arm number.

**Figure 9:**
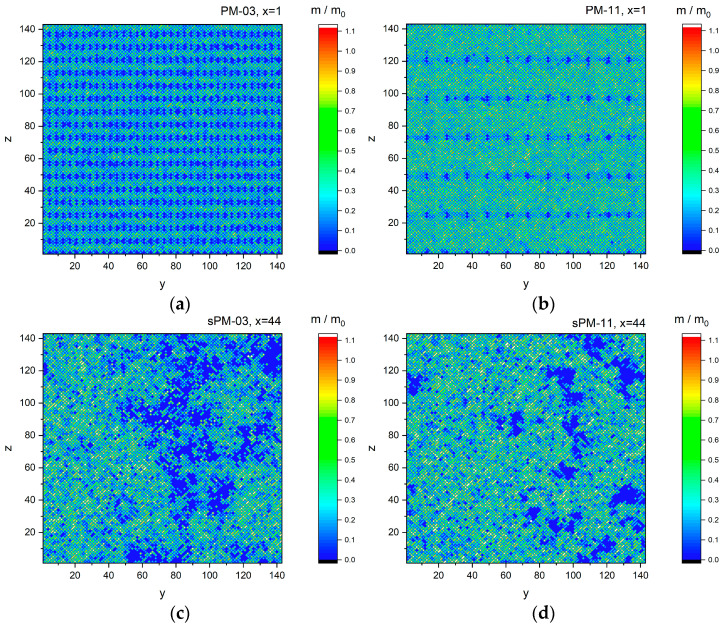
Reduced mobility for (**a**) PM-03, (**b**) PM-11, (**c**) sPM-03, and (**d**) sPM-11. The cross section number is also indicated in the heading.

Thus, it can be seen that all the simulation results analyzed above indicate that the local structure of the polymer material determines the solvent dynamics. This raises the question of what solvent mobility looks like at a non-local scale and how it is spatially distribution-dependent. Mobility was calculated as a ratio of successful movements to total movement trials in a given point of space and time-averaged over the entire simulation run. Figure 9a–d present the reduced mobility of solvent motion (relative to a system consisting of the solvent alone) in regular PM and irregular sPM networks, characterized by different mesh size values. The data presented are for selected in-plane cross sections. It can be seen that, in the case of regular networks, in principle, the obstructive effect of the network on the mobility of the solvent results in a strong slowdown of the solvent molecules in the nearest layers near the branching points of the polymer network, regardless of the mesh size (Figure 9a,b). Irregular networks (Figure 9c,d) show a large clustering of points with highly reduced movement (relative mobility below 0.1), consisting of slower polymer units and solvent molecules apparently arrested nearby. An increase in the mesh size leads to a significant decrease in the number of sites with a very low probability of motion. It should also be noted that the spectrum of solvent movement probability values is much wider for sPM networks than for regular PM networks.

## 4. Conclusions

In this work, the effect of structure in complex polymer systems on solvent dynamics was studied. These types of polymeric materials are characterized by disorder, whereas typically only regular systems are theoretically studied. Therefore, a coarse-grained model concerning irregular networks and star-shaped macromolecules with an explicit solvent was designed. Monte Carlo simulations were carried out using the Dynamic Lattice Liquid (DLL) algorithm based on the concept of cooperative motions in dense soft-matter systems. This tool allows for the study of very large systems (consisting of more than 10^6^ elements) on long time scales. The DLL algorithm made it possible to both simulate the synthesis of the polymeric materials and study the solvent dynamics after the reaction was finished. In this work, the dynamics of solvent molecules in regular and irregular polymer networks, as well as in solution of star-like polymers (modeling separate network nodes), was compared.

The self-diffusion coefficients and diffusion relaxation times of the solvent were determined, showing that the differences in these parameters were only quantitative and that the influence of irregular networks appeared to be significantly stronger than that of regular networks and star-like polymers. It also turned out that despite the low polymer concentration, between 2% and 15%, the appearance of anomalous diffusion was observed in the systems studied. Deviations from normal diffusion were found to be small and strongly dependent on the mesh size of the polymer network. The variation in the dynamics of the solvent was shown to depend on the position of the solvent molecule in relation to regions of higher or lower polymer concentration. The movement of the solvent inside and near the polymer can be described as subdiffusive precisely for short time scales for bulk solvent. The release of the solvent molecule from a region of locally high polymer concentration led to a significant instantaneous acceleration of its motion. Thus, the influence of the local structure rather than the averaged or model-regular structure of the cross-linked and branched macromolecular systems on the dynamics of the surrounding and penetrating solvent should be emphasized.

## Data Availability

The original contributions presented in the study are included in the article, further inquiries can be directed to the corresponding author.
